# Features of toll-like receptor genes (TLR-2, TLR-3, TLR-4 and TLR-6) polymorphism in open-angle glaucoma patients

**DOI:** 10.18699/vjgb-25-15

**Published:** 2025-02

**Authors:** A.V. Shevchenko, V.F. Prokofiev, V.I. Konenkov, V.V. Chernykh, A.N. Trunov

**Affiliations:** Research Institute of Clinical and Experimental Lymрhology – Branch of the Institute of Cytology and Genetics of the Siberian Branch of the Russian Academy of Sciences, Novosibirsk, Russia; Research Institute of Clinical and Experimental Lymрhology – Branch of the Institute of Cytology and Genetics of the Siberian Branch of the Russian Academy of Sciences, Novosibirsk, Russia; Research Institute of Clinical and Experimental Lymрhology – Branch of the Institute of Cytology and Genetics of the Siberian Branch of the Russian Academy of Sciences, Novosibirsk, Russia; Novosibirsk Branch of the S.N. Fedorov National Medical Research Center “MNTK “Eye Microsurgery” of the Ministry of Health of the Russian Federation, Novosibirsk, Russia; Novosibirsk Branch of the S.N. Fedorov National Medical Research Center “MNTK “Eye Microsurgery” of the Ministry of Health of the Russian Federation, Novosibirsk, Russia

**Keywords:** primary open-angle glaucoma, POAG, polymorphism of toll-like receptor genes, TLR, linkage disequilibrium, первичная открытоугольная глаукома, ПОУГ, полиморфизм генов толл-лайк рецепторов, TLR, неравновесное сцепление

## Abstract

Modern research shows that innate immunity plays an important role in the pathogenesis of primary open-angle glaucoma (POAG). An increase in the content of toll-like receptors (TLR) in the glaucomatous retina of the human eye was revealed. TLRs can modulate the immune response in glaucoma; provide early recognition of damaging agents, activation of signaling pathways and effector mechanisms of the nonspecific immune defense system aimed at restoring homeostasis. The TLR-encoding genes’ polymorphism alters the amino acid structure of the receptors, which leads to changes in their immune functions: expression level, ligand-binding and coreceptor functions, transport and signal transmission. The aim was to analyze the association of the TLR2 (rs5743708), TLR3 (rs3775291), TLR4 (rs4986790, rs4986791) and TLR6 (rs5743810) polymorphisms with primary open-angle glaucoma in patients of Western Siberia. Methods: 99 patients (52 men and 47 women) with a diagnosis of primary open-angle glaucoma were examined. The comparison group consisted of 100 people (81 women and 19 men). TLR2 (rs5743708), TLR3 (rs3775291), TLR4 (rs4986790, rs4986791) and TLR6 (rs5743810) polymorphisms were analyzed by RT-PCR using test systems with Syber Green (Lytex, Russia). Statistical analysis was performed using the software package SPSS 23.0 and Arlequin 3.5.2.2. Results: the distribution of genotypes in the patient group and in the control group corresponded to the Hardy–Weinberg equilibrium. The genotype frequencies did not significantly differ between the two analyzed groups. The frequency of TLR2-753 ArgArg:TLR6-249 ProPro was increased in the group of patients with POAG. The linkage disequilibrium between two polymorphic positions of the TLR4 gene was revealed. In addition, the linkage disequilibrium between TLR2-TLR6 gene for the glaucoma group and the control group was revealed. Conclusion: an increase in certain genotypes in the patient group relative to the control group may indirectly indicate the involvement of infectious factors in the initiation of POAG. However, despite the proven importance of the participation of their protein products in the pathogenesis of glaucoma, the relationship of TLR polymorphism requires additional research taking into account the ethnic characteristics of patients and intergenic interactions for a better understanding of the complex mechanisms of disease development. This will help carry out early diagnosis and develop the necessary therapeutic strategy.

## Abstract (ru)

Современные исследования показывают, что в патогенезе первичной открытоугольной глаукомы (ПОУГ) важную роль играет врожденный иммунитет. Выявлено повышение содержания толл-лайк рецепторов (TLR) в глаукоматозной сетчатке глаза человека. TLR могут модулировать иммунный ответ при глаукоме, обеспечивают раннее распознавание повреждающих агентов, активацию сигнальных путей и эффекторных механизмов системы неспецифической иммунной защиты, направленных на восстановление гомеостаза. Полиморфизм кодирующих TLR генов влияет на аминокислотную структуру рецепторов, приводя к изменению лигандсвязывающей и корецепторной функции, транспортировку и передачу сигналов. Целью работы был анализ ассоциированности полиморфизма генов TLR2 (rs5743708), TLR3 (rs3775291), TLR4 (rs4986790, rs4986791), TLR6 (rs5743810) с первичной открытоугольной глаукомой у пациентов Западной Сибири. Обследовано 99 пациентов (52 мужчины и 47 женщин) с диагнозом первичной открытоугольной глаукомы. Группу сравнения составили 100 человек (81 женщина и 19 мужчин). Полиморфизм генов TLR2 (rs5743708), TLR3 (rs3775291), TLR4 (rs4986790, rs4986791), TLR6 (rs5743810) анализировали методом РТ-ПЦР с использованием коммерческих тест-систем с интеркалирующим красителем Syber Green (Lytex, Россия). Статистический анализ проводился с использованием программного пакета SPSS 23.0 и Arlequin 3.5.2.2. Показано, что распределение полиморфных маркеров в группе пациентов и в контрольной группе соответствовало равновесию Харди–Вайнберга. Их частоты между двумя анализируемыми группами достоверно не различались. Частота TLR2-753 ArgArg:TLR6-249 ProPro была повышена в группе пациентов с ПОУГ. Выявлено неравновесное сцепление между двумя полиморфными позициям гена TLR4. Кроме того, выявлено нарушение равновесия между парами генов TLR2-TLR6 для группы с глаукомой и контрольной группы. Повышение определенных генотипов в группе пациентов относительно контрольной группы может косвенно свидетельствовать об участии инфекционных факторов в инициации ПОУГ. Однако связь полиморфизма TLR генов, несмотря на доказанную значимость участия их белковых продуктов в патогенезе глаукомы, требует дополнительных исследований с учетом этнических особенностей пациентов и межгенных взаимодействий для лучшего понимания сложных механизмов развития заболевания. Это поможет проводить раннюю диагностику и разрабатывать необходимую терапевтическую стратегию.

## Introduction

Primary open-angle glaucoma (POAG) is the multifactorial disease leading to progressive and irreversible vision loss, is currently a serious medical problem, including due to insufficiently studied mechanisms of damage to the optic nerve and death of retinal ganglion cells (Baudouin et al., 2021; Tezel, 2022). Modern research shows that innate immunity plays an important role in the pathogenesis of POAG. It has been established that the inducers of inflammation at the cellular level in glaucoma are the molecular structures of DAMPs (damage associated molecular patterns) released from the tissue membranes of the eye when they are damaged, including those formed as a result of an increase in intraocular pressure level (IOP) (Tezel, 2022). Excessive accumulation of DAMPs is identified by cellular pattern-associated receptors (PRRs), which are located on endosomal membranes and in the cytoplasm. It has been shown that with the development of POAG, PRRs provide early recognition of damaging agents, activation of signaling pathways and effector mechanisms of the nonspecific immune defense system aimed at restoring homeostasis (Luo et al., 2010).

The most well-studied family of PRRs are Toll-like receptors (TLRs), the expression of which has been detected in all membranes of the human eye (Stewart et al., 2015). Proteomic and immunohistochemical studies have shown an increase in TLR expression in the human glaucomatous retina, indicating that TLRs can modulate the immune response in glaucoma (Luo et al., 2010; Titi-Lartey et al., 2022). To date, two groups of functionally different TLRs have been identified in humans: transmembrane, which include TLR1, TLR2, TLR4, TLR5, TLR6 and TLR11, and intracellular – TLR3, TLR7, TLR8, TLR9. It has been shown that polymorphism of TLR-encoding genes affects the amino acid structure of receptors, leading to changes in their expression level, ligand-binding and coreceptor functions, and signal transport and transmission. In addition, the features of the functions are related to the location of the polymorphic TLR site. Polymorphism of loci encoding the extracellular domain of the receptor may additionally lead to a change in binding affinity and subsequent immune response, whereas mutations in the cytoplasmic domain of TLR may lead to a change in downstream signaling, despite normal binding (Törmänen et al., 2017; Macedo et al., 2019; Zhang et al., 2021). The aim of our research is to analyze the association of TLR2 (rs5743708), TLR3 (rs3775291), TLR4 (rs4986790, rs4986791), TLR6 (rs5743810) gene polymorphisms with primary open-angle glaucoma in patients of Western Siberia

## Materials and methods

Patients. 99 patients with diagnosed stage II primary openangle glaucoma were examined – 52 (52.53 %) men and 47 (47.47 %) women. The average age of the patients was 62.8 ± 4.3 years. The diagnosis was established on the basis ophthalmological examination (determination of visual acuity, binocular ophthalmoscopy, spheroperimetry, echophthalmography, optical coherence tomography, measurement of intraocular pressure). The criteria for diagnosis were: a pronounced change in the field of vision in the paracentral region, a narrowing of the field of vision from the nose in the upper or lower nasal segment by more than 10 degrees relative to normal values, but not less than 15 degrees from the fixation point; the marginal nature of the deepening of the optic nerve. Patients of the main group had compensated (<22 mmHg (against the background of drug therapy)) or moderately elevated (<33 mmHg) intraocular pressure. The comparison group consisted of 100 people – 81 women and 19 men. The average age was 63.5 ± 0.4 years. The criterion for inclusion in the comparison group was the absence of a diagnosis of glaucoma in the subjects.

Both groups of patients did not significantly differ in age characteristics. The patients of both groups were representatives of the phenotypically Caucasian population of Russia, who were born in this territory, identifying themselves and their forebearers as “Russians”. The exclusion criteria for both groups were: acute chronic inflammatory diseases of the visual organ and their exacerbations, the presence of diabetic retinopathy, neovascular glaucoma, uveitis of various etiologies and localization, hemophthalmos, autoimmune and tumor processes of any localization, diabetes mellitus without ophthalmological manifestations. The study was approved by the Committees on Biomedical Ethics of the Scientific Research Institute of Clinical and Experimental Lymphology, a branch of the Institute of Cytology and Genetics of the Siberian Branch of the Russian Academy of Sciences (Protocol No. 177 dated 02.02.2003) and the Novosibirsk Branch of FSAI “The academician S.N. Fyodorov Federal State Institution Intersectoral Research and Technology Complex ‘Eye Microsurgery’” of the Ministry of Health of the Russian Federation (Protocol No. 2 dated 02.09.2018). Informed consent was obtained from all patients for blood collection, as well as for the use of research data for scientific purposes

DNA isolation and genotyping. Genomic DNA was obtained from whole blood samples taken for EDTA using the phenol chloroform method. Single nucleotide polymorphism (SNP) of the TLR2 (rs5743708), TLR3 (rs3775291), TLR4 (rs4986790, rs4986791), TLR6 (rs5743810) genes was detected by real-time polymerase chain reaction (RT-PCR) using commercial test systems with intercalating dye Syber Green (Lytex, Russia) in accordance with the manufacturer’s instructions.

Statistical analysis. The “case-control” scheme was used in the study. The distribution of polymorphic markers in the patient group and the control group was checked for compliance with the Hardy–Weinberg equilibrium (HWE) using the chi-square criterion. The frequency differences were determined using a two-way Fisher precision test. A p < 0.05 was considered statistically significant. If the null hypotheses were not confirmed at a given level of significance α = 0.05, then in cases of multiple comparisons, the adjusted value of p was determined using the Bonferroni correction calculated by the one-step method (Narkevich et al., 2020). Odds ratios (OR) were calculated with a 95 % confidence interval (CI). The analysis of nonequilibrium coupling was carried out by the maximum likelihood analysis method. All statistics were carried out using the software package SPSS 23.0 and Arlequin 3.5.2.2.

## Results

We analyzed polymorphic variants of the coding regions of the TLR2 (rs5743708), TLR3 (rs3775291), TLR4 (rs4986790, rs4986791), TLR6 (rs5743810) genes in a group of patients with primary open-angle glaucoma II (advanced) stage relative to the control group. The distribution of polymorphic markers in the patient group and in the control group corresponded to the Hardy–Weinberg equilibrium (Table 1).

**Table 1.**
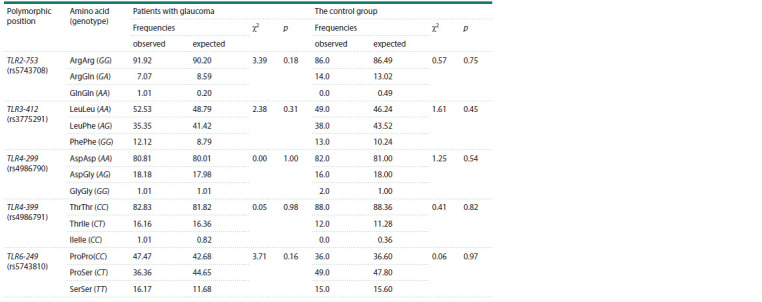
Correspondence of the frequencies of polymorphic markers to the Hardy–Weinberg equilibrium in the group of glaucoma patients and the control group

The frequency distribution in the positions analyzed by us did not significantly differ between the two groups (Table 2). Assuming that the presence of features of complex network interactions of protein products of the genes we study is the reflection of their genetic structure, we analyzed the differences in the complexes of genotypes in two groups. We identified a single TLR2-753 ArgArg:TLR6-249 ProPro complex, the frequency of which was increased in the group of patients with POAG (OR = 1.84, p = 0.0425, p_cor = 0.297).

**Table 2.**
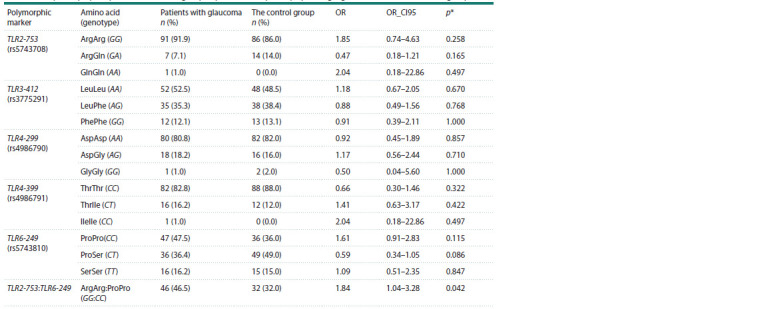
Analysis of polymorphic markers in the group of patients with primary open-angle glaucoma and in the control group Notе. OR_CI95 is the 95 % confidence interval for OR, p* is the level of statistical significance of differences according to the exact Fisher method (twosided).

Since the TLR4 polymorphic positions analyzed by us are located in one exon of the gene, and the polymorphic loci of the TLR2, TLR3, TLR6 genes are on the same chromosome we analyzed the linkage disequilibrium (LD) of these positions. The characteristics of the analyzed single nucleotide positions of TLR genes are given in Table 3. The frequency of the minor allele in most of the loci analyzed by us was more than 5 %, with the exception of rs5743708 of the TLR2 gene

**Table 3.**
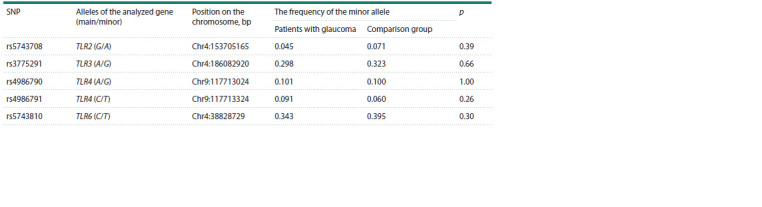
Characteristics of single nucleotide positions Notе. Position is the distance from the telomeres of the short arm of the chromosome, bp – base pair.

We have revealed the linkage disequilibrium between two polymorphic positions of the TLR4 gene (Table 4). The analysis of multiple SNPs showed that the most common haplotype for the TLR4 rs4986790 and rs4986791 SNPs is A/C for both groups, the A/T haplotype is completely absent in the comparison group. The Lewontin’s D′ coefficient between SNP rs4986790 and rs4986791 are 0.8146 in the patient and 1.0000 in the comparison group. In addition, we found the linkage disequilibrium between the TLR2-TLR6 genes (D′ = 0.6615 and D′ = 0.5277 for the glaucoma group and the control group, respectively). For the TLR3-TLR6 genes, D′ = 0.1997 and D′ = 0.2008 in the glaucoma group and the control group, respectively. At the same time, the analysis of haplotype frequencies between the groups did not reveal any significant differences.

**Table 4.**
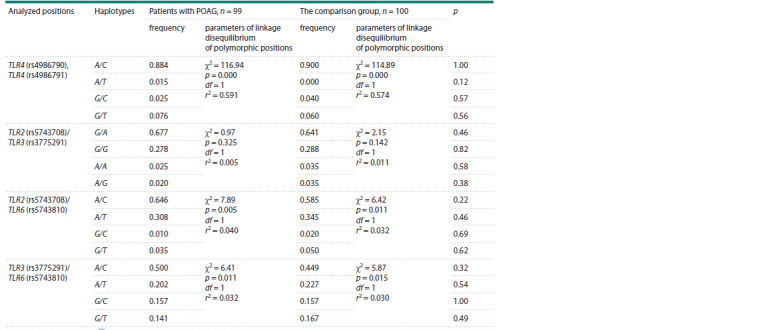
Haplotype frequencies and parameters of the linkage disequilibrium between the analyzed polymorphic loci Notе. df – degree of freedom, r2 – correlation coefficient.

## Discussion

Open-angle glaucoma is considered as a multifactorial disease with convincing evidence of the involvement of the genetic component in its development. To date, studies of genetic associations have identified many loci that contribute to the genetic risk of developing POAG. TLRs are important factors of the innate immune system; however, the results of the study concerning the association of TLR polymorphism with the disease are quite contradictory

It is known that TLR2 is a mediator of retinal degeneration in response to oxidative stress, functions as a “bridge” between oxidative damage and complement-mediated retinal pathology and is associated with the development of a number of ophthalmopathologies (Mulfaul at al 2020; Titi-Lartey et al., 2022). It has been shown that the p.Arg753Gln missense mutation leads to a deficiency in TLR2 signaling due to impaired TLR2-TLR6 heterodimerization, tyrosine phosphorylation and further cascade, without affecting TLR2 expression (Xiong at al., 2012). However, we did not identify the association of TLR2 and TLR6 polymorphisms in the analyzed positions with the development of POAG. Similar results have been shown by Japanese researchers for TLR2 (Nakamura et al., 2009). We did not find any data on the polymorphism of the TLR6 gene in glaucoma in the literature. At the same time, the analysis of the Pro249Ser marker and the construction of a three-dimensional model for TLR6 revealed conformational changes in the structure of the mutant protein, presumably affecting the binding of ligands and receptors: in the wild type, binding pockets near proline (Pro) are larger in volume, whereas in the mutant one, the walls of the pockets are located close to each other. This significantly affects the ability of the mutant protein to enter into significant interactions, since it is known that most binding regions and active sites are located in the largest pocket cavity. In addition, the wild-type protein, being more flexible, has more possibilities for ligand-induced movements, whereas in the mutant ligand, induced movement is limited only by side chain rearrangements. In addition, the mutant protein is less stable. All this confirms that TLR6 polymorphism affects the structure and functionality of the protein (Hamann et al., 2013; Semlali et al., 2018). Considering that TLR2 and TLR6 function during the formation of a heterodimer, we analyzed their complex polymorphism during the development of POAG and found that carriers of the homozygous wild-type genotype TLR2-753 ArgArg:TLR6-249 ProPro have a higher chance of developing the disease, which may be explained precisely by the peculiarities of joint functioning during ligand recognition and stimulation of further immune cascade. In addition, since the TLR2 and TLR6 genes are within the same chromosome, we performed an analysis of the linkage disequilibrium and showed a change of the LD positions of the TLR2-TLR6 genes analyzed. This means that certain alleles of two genes may appear in a single haplotype more often than would be expected with a random combination. Previously, the linkage disequilibrium for these polymorphic positions has been shown in other studies (Stashkevich et al., 2022). At the same time, we have not revealed any differences in the frequencies of haplotypes.

The relationship of TLR3 gene polymorphism in the analyzed position is also shown for a number of ophthalmopathologies (Titi-Party et al., 2022). But stratification analysis by ethnicity indicates that rs3775291 is associated, in particular, with all forms of macular degeneration only in Caucasians, but not in East Asians (Ma et al., 2016). The polymorphism of Leu412Phe affects the normal dimerization of TLR3, which leads to a change in protein activity necessary for proper signaling (Ranjith-Kumar et al., 2007). In glaucoma, TLR3 and TLR4 have been shown to initiate nephroptosis – the regulated proinflammatory lytic form of necrotic cell death characterized by cell swelling followed by rupture of the plasma membrane with the release of cellular contents (Basavarajappa et al., 2023). The activity of TLR3 involved in the recognition of nucleic acids released from damaged cells is mainly associated with the early stage of glaucoma (Soto, Howell, 2014). However, the association of polymorphism of this gene with glaucoma is controversial in the literature. Several studies of the WDR36 locus of the TLR3 gene, including SNP rs3775291, have demonstrated its role as a modifier gene in POAG due to the clinical severity of the process (Hauser et al., 2006; Meer et al., 2021). However, a meta-analysis of 122 publications did not confirm the significant role of this polymorphic position in the genetic predisposition to POAG or its subtypes. At the same time, the authors are inclined to believe that further research is needed in specific populations (Liu et al., 2017).

rs4986790 and rs4986791 polymorphisms in exon 3 of the TLR4 gene are among the most well-known and frequently studied SNPs. Polymorphism in these positions leads to changes in the polypeptide chains of the extracellular domain of the receptor and affects binding to the coreceptor, which leads to hyperactivity of the receptor. This can cause dysfunction of the TLR4 molecule and disrupt the host’s immune system (Arbour et al., 2000; Jahantigh et al., 2013; Lin et al., 2019). Currently, the results of meta-analyses of the association of these SNPs with POAG by different research groups indicate that the data differ in different ethnic groups and further research is needed (Chaiwiang, Poyomtip, 2019; Lin et al., 2019). At the same time, almost all studies have shown linkage disequilibrium of TLR4 rs4986790 and TLR4 rs4986791 (Guimarãesa et al., 2018; Kania et al., 2022), which is confirmed in our study. This indicates that the recombination of the chromosome regions on which these polymorphic markers are located is inherited as a single block. It is believed that it is the analysis of haplotypes in the presence of a high degree of multilocus LD that can significantly increase the statistical significance of the study (Jiang, et al., 2014); however, we have not revealed significant differences in the analyzed frequencies of the TLR4 gene haplotypes

Since the receptors of TLR1, 2, 4, 5, 6 and 10 belong to surface membrane receptors that recognize mainly lipid components of bacterial structures, and TLR3, 7, 8 and 9 are expressed on the membranes of intracellular organelles, ligands for which are components of nucleic acids of viruses (Akira et al., 2001; Sameer, Nissar, 2021), an increase in the frequency of a number of TLR gene genotypes in a group of patients may indirectly indicate the involvement of infectious factors in the initiation of POAG.

## Conclusion

Thus, the relationship of TLR gene polymorphism, despite the proven importance of the participation of their protein products in the pathogenesis of glaucoma, requires additional research taking into account the ethnic characteristics of patients. In addition, it is necessary to take into account gene-gene interactions to better understand the complex mechanisms of disease development, which will help to carry out early diagnosis and develop the necessary therapeutic strategy.

## Conflict of interest

The authors declare no conflict of interest.
